# An estimate of pocket closure and avoided needs of surgery after scaling and root planing with systemic antibiotics: a systematic review

**DOI:** 10.1186/1472-6831-14-159

**Published:** 2014-12-22

**Authors:** Mirela Kolakovic, Ulrike Held, Patrick R Schmidlin, Philipp Sahrmann

**Affiliations:** Clinic of Preventive Dentistry, Periodontology and Cariology, Center of Dental Medicine, University of Zurich, Plattenstrasse 11, 8032 Zurich, Switzerland; Horten Center, University Hospital Zurich, Pestalozzistrasse 24, 8091 Zurich, Switzerland

**Keywords:** Periodontitis, Antibiotics, Treatment needs, Non-surgical therapy, Amoxicillin, Metronidazole

## Abstract

**Background:**

Relevant benefits of adjunctive medication of antibiotica after conventional root surface debridement in terms of enhanced pocket depth (PD) reduction have been shown. However, means and standard deviations of enhanced reductions are difficult to translate into clinical relevant treatment outcomes such as pocket resolution or avoidance of additional surgical interventions. Accordingly, the aim of this systematic review was to calculate odds ratios for relevant cut-off values of PD after mechanical periodontal treatment with and without antibiotics, specifically the combination of amoxicilline and metronidazol, from published studies. As clinical relevant cut-off values “pocket closure” for PD ≤ 3mm and “avoidance of surgical intervention” for PD ≤ 5 mm were determined.

**Methods:**

The databases PubMed, Embase and Central were searched for randomized clinical studies assessing the beneficial effect of the combination of amoxicillin and metronidazole after non-surgical mechanical debridement. Titles, abstracts and finally full texts were scrutinized for possible inclusion by two independent investigators. Quality and heterogeneity of the studies were assessed and the study designs were examined. From published means and standard deviations for PD after therapy, odds ratios for the clinically relevant cut-off values were calculated using a specific statistical approach.

**Results:**

Meta-analyses were performed for the time points 3 and 6 month after mechanical therapy. Generally, a pronounced chance for pocket closure from 3 to 6 months of healing was shown. The administration of antibiotics resulted in a 3.55 and 4.43 fold higher probability of pocket closure after 3 and 6 months as compared to mechanical therapy alone. However, as the estimated risk for residual pockets > 5 mm was 0 for both groups, no odds ratio could be calculated for persistent needs for surgery. Generally, studies showed a moderate to high quality and large heterogeneity regarding treatment protocol, dose of antibiotic medication and maintenance.

**Conclusion:**

With the performed statistical approach, a clear benefit in terms of an enhanced chance for pocket closure by co-administration of the combination of amoxicillin and metronidazole as an adjunct to non-surgical mechanical periodontal therapy has been shown. However, data calculation failed to show a benefit regarding the possible avoidance of surgical interventions.

## Background

Periodontitis is a widespread inflammatory disease of the tooth-supporting soft and hard tissues [1–3] with an intermittent destruction process. It progresses either chronically or aggressively [4], but in either case, bacterial involvement in biofilms is regarded as the primary etiologic factor for both disease initiation and progression [5, 6]. Accordingly, the pivotal aim of cause-related periodontal therapy is based on the removal of the pathogenic microbial challenge and the successful prevention of its re-establishment in the ecological niches [7]. Clinically, this is achieved by mechanical debridement using scalers, curettes and/or ultrasonic instruments along with proper oral hygiene instruction [8, 9]. In this context, however, a complete root surface cleaning has been shown to be an unrealistic aim: Especially in pockets exceeding a depth of 6 mm, a perfect debridement is impossible - even when performed by experienced operators [10, 11]. Despite these technical limitations, relevant outcome parameters like depth and number of pockets can be significantly reduced and maintained irrespective of the initial probing depth [12, 13]. However, in many situations periodontitis is not completely resolved by non-surgical mechanical means alone [14], especially in difficult to clean areas such as multi-rooted teeth and complex bone defect configurations [15].

Thus, the use of antimicrobials is a viable approach to improve the clinical outcomes. The adjunctive administration of systemic antibiotics for instance has been shown to offer special healing benefits to improve the mechanical debridement in critical sites [16]. In addition, periopathogenic bacteria are known to colonize not only subgingival tooth surfaces but also hide in oral niches like deep plications of the tongue, crypts of the palatopharyngeal tonsils or the inner buccal mucosa and its recesses, where they are mostly out of the reach of mechanical treatment [17, 18]. Noteworthy, some bacteria were even shown to invade periodontal soft tissue cells [19–21], where they remain inaccessible for conventional mechanical debridement as well. Therefore, antibiotic therapy has gained a long tradition in periodontitis therapy [22]. However, well-controlled studies are limited to specific agents [23], among which, amoxicillin, metronidazole and their combination being the most frequently investigated antibiotics [24]. To date, a considerable number of studies have consistently shown a superiority of the systemic administration of these agents together with scaling and root planning (SRP), mainly in terms of probing pocket depths (PPD), clinical attachment levels (CAL) and changes as compared to SRP alone [24]. However, the problem of adverse side-effects and especially a seemingly ever increasing risk of bacterial resistance [25] urge clinicians to balance risks and benefits well with each individual patient.

Among parameters for oral hygiene, marginal inflammation and gingival recession, periodontal pocket depth (PPD) and clinical attachment loss (CAL) are still the most important surrogate parameters for clinical changes. Whereas CAL indicates the amount of periodontal destruction that will not necessarily be recovered in most cases with successful periodontal treatments, PPD is the parameter that should improve significantly during therapy. As PPD values up to 3 mm are regarded as being compatible with periodontal health, pockets exceeding 5 or 6 mm might not align with immediate treatment success or long-term stability. As these pockets show a significantly enhanced risk for further bacterial regrowth and attachment loss [26], they constitute an indication for additional – in most cases surgical - treatments. This fact is well reflected in the cut-off values for pocket depths of the Community Periodontal Treatment Index of Treatment Needs (CPITN) and the Periodontal Screening Record (PTR) [27, 28]. hoo.de > ly dation section tge language t in its te added. the phrase into. “ text as follows: ults in enhanced heterogeneit.

Following the guidelines for the conduction of the respective studies, systematic reviews with meta-analyses present differences of various treatment modalities expressed as means and standard deviations of the above-mentioned outcome parameters (e.g. PPD and/or CAL) in millimeters [29–31]. Despite being statistically flawless, this mode of data presentation renders it difficult for clinicians and patients to estimate the clinical benefit in terms of an adjunctive treatment [32], as direct information on the degree of clinical success rate is not provided. Regarding a clinically applicable success estimation after periodontal treatment, the reduction of the periodontal pocket depth on a physiologic level of up to 3 mm, i.e. the clinical pocket closure, remains the most important end parameter. Beyond that, a further distinction between sites with moderately enhanced pocket depths that might remain stable over long time periods and those, which most probably need further invasive therapy, seems reasonable [26]. Thus, distinct cut-off values of ≤ 3 mm and ≤ 6 mm PPD might constitute important landmarks to clinicians and patients for every day decision-making.

Therefore, it was the aim of the present study to conduct a meta-analysis based on data of the existing literature on combined administration of amoxicillin and metronidazole as an adjunct to SRP, calculating the probability of clinical success by using these relevant cut-off values of 3 an 5 mm PPD to provide estimated for pocket closure and avoidance of surgery after scaling and root planing with systemic antibiotics.

## Methods

This study was planned and conducted in accordance to the PRISMA guidelines for systematic reviews [29]. Modifications were made with regard to the study specific presentation of the outcomes expressed as means and standard deviations instead of estimated probability for the cut-off values.

The focused question according to the PICO criteria was:

“What is the outcome after non-surgical subgingival debridement with or without systemic administration using a combination of amoxicillin and metronidazole in healthy humans with chronic or aggressive periodontitis in terms of the estimated odds ratio for pocket closure (i.e. PPD ≤ 3 mm) or avoidance of surgery (i.e. PPD ≤ 5 mm)?”

A meta-analysis was conducted for data at 3 and 6 month after intervention.

### Search strategy

**Figure 1:**
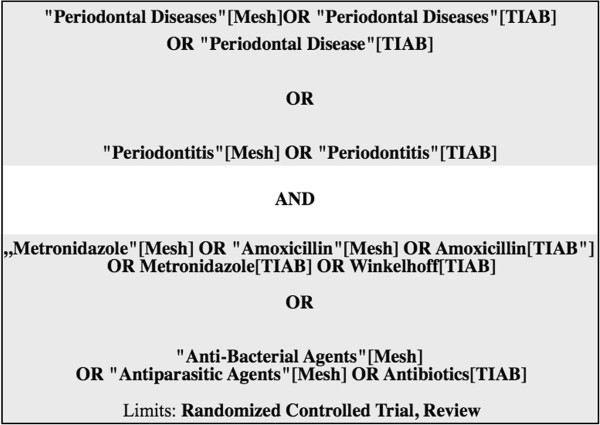
**Search items for the electronic literature search.** MeSH – Mdical Subject Headings, TIAB – Title and Abstract.

**Figure 2:**
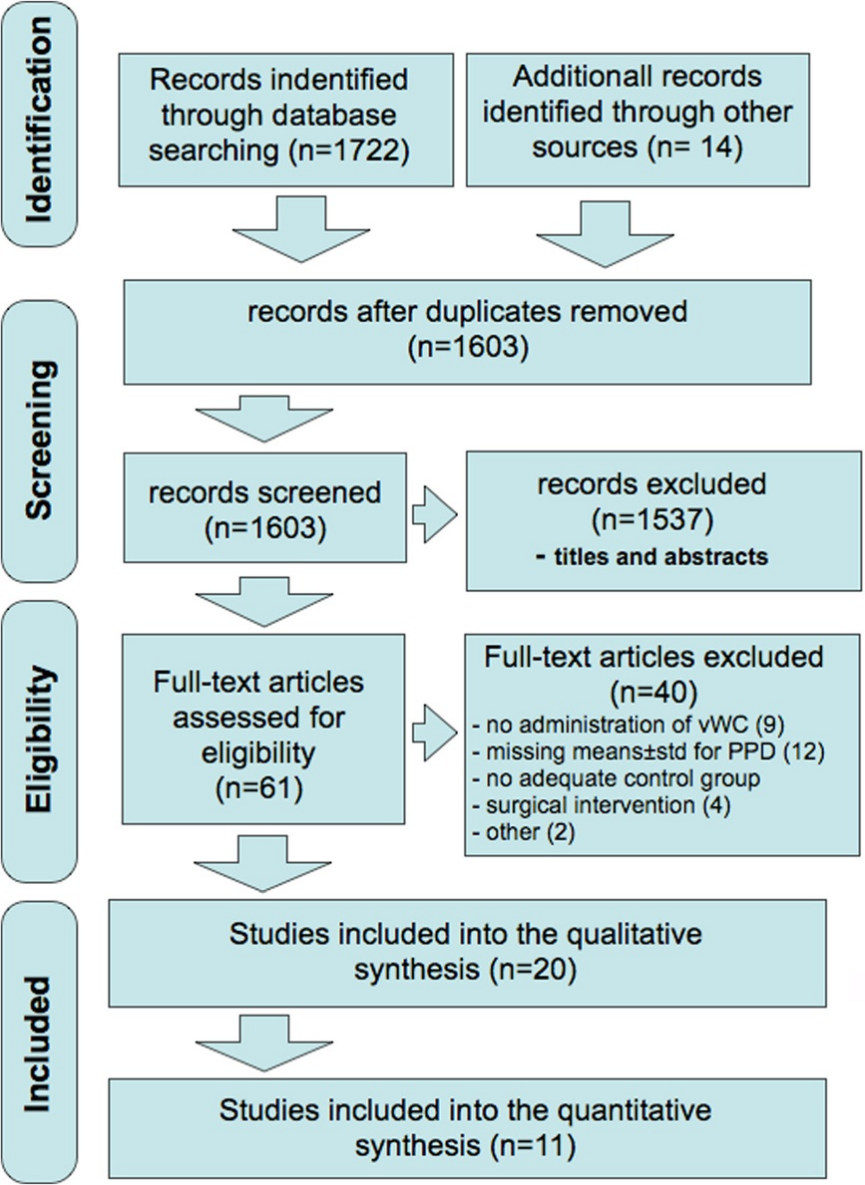
**Screening strategy performed by two independent reviewers.** vWC – vanWinkelhoff Cocktail.

A literature search up to June 2013 was conducted in the US National Library of Medicine (PubMed), the Exerpta Medical Database (Embase) and the Cochrane Central Register of Controlled trials (CENTRAL) using the search terms and combinations presented in Figure 1. After title and abstract screening, an additional hand search was performed in the reference lists of all full texts of interest and the index of contents of *Journal of Clinical Periodontology*, *Journal of Periodontal Research* and *Journal of Periodontology*. The search was conducted without language restriction. The literature search was performed by two independent reviewers (Kolakovic and Sahrmann). In case of discrepancies, study exclusion was determined after discussion. The search strategy is depicted in Figure 2.

### Eligibility criteria

In order to include data from studies of highest quality, only randomized controlled clinical trials were considered. Studies comparing the clinical outcomes of non-surgical periodontal treatment with and without adjunctive systemic antibiotic therapy focussing on the combination of amoxicillin and metronidazole, in otherwise healthy patients were included. Studies had to report data for periodontal probing depths after a time interval of at least 3 months after treatment, presented as means and standard deviations, which displayed normal data distribution. Studies on patients with known diseases or drug intake that potentially affects progression and therapy of periodontitis (diabetes, immunosuppressive medication etc.) were excluded. In order not to exclude an entity that is often specifically treated with a concomitant antibiotic medication smokers were not excluded.

### Assessment of heterogeneity

To assess the comparability of the selected studies, data on diagnosis, patient populations, exclusion criteria, treatment protocols including pre-treatment, interventions and maintenance protocols of each study were extracted.

### Quality assessment

To estimate the potential bias of the different studies included, the described method of randomization, the concealment strategy of the allocation and the blinding of the operator performing the clinical examination were assessed.

### Statistical analysis

From each study, we extracted the number of participants and the mean pocket depth and standard deviation at the follow-up examination(s). If not exactly described in the respective statistical methodology section in the original paper, we assumed that the pocket depths were normally distributed if they were presented as mean and standard deviation. Based on available data sets, the probability of clinical success, expressed as the proportion of pockets < 3 mm, and the proportion of persisting pockets > 3 mm and > 5 mm, respectively, using the method proposed by Hauri and co-workers [32] was determined. For this purpose, the odds ratios (OR) and their 95% confidence intervals from the derived event rates in experimental and control group for each of the studies were calculated. For pooling of these ORs a fixed effects meta-analysis model was used. All analyses were performed with R, a free software environment for statistical computing and graphics [33].

## Results

### Study selection

The electronic literature search provided 1603 potentially includable studies. Based on titles and abstracts, 1537 of these were excluded (93% agreement between reviewers prior to discussion). Based on the full text assessment further 40 studies were excluded due to administration of antibiotics other than the combination of interest, data presentation without means and standard deviations or inadequate intervention in either test or control group (see Figure 2 and Table 1[34–58]). In case of missing clinical data or unsuitable data presentation the corresponding authors were contacted via electronic mail requesting further information, [59, 60]. If no reply was received within 12 weeks, the respective study had to be excluded. The remaining studies could not be included into the meta-analysis due to their individual time points of data evaluation.

**Table 1:** **Excluded studies**

Excluded studies	Reason for exclusion	Reason group
Akincibay 2008 [34]	Doxicyclin in the control group	2
Carvalho 2005 [35]	No PPD data presentation as means ± standard deviation	1
Cionca 2010 [36]	No PPD data presentation as means ± standard deviation	1
Ehmke 2003 [37]	No PPD values given before and after treatment	1
Ehmke 2005 [38]	No PPD data presentation as means ± standard deviation	1
Eickholz 2013 [39]	no adequate control group	2
Eisenberg 1991 [40]	short term evaluation (after 3 weeks)	5
Flemmig 1998 [42]	No PPD data presentation as means ± standard deviation	1
Griffiths [55]	no adequate control group	2
Guerrero [34]	No PPD data presentation as means ± standard deviation	1
Haffajee [44]	Administration of metronidazole only	3
Haffajee 2008 [43]	Administration of metronidazole only	3
Hartmann 1986 [44]	Administration of metronidazole only	3
Hernandez 1987 [45]	No PPD data presentation as means ± standard deviation	1
Jenkins 1989 [46]	no adequate control group	2
Joyston 1984 [47]	Administration of metronidazole only	3
Joyston 1986 [48]	Administration of metronidazole only	3
Lindhe 1982 [49]	no adequate control group	2
Loesche 1987 [50]	Administration of metronidazole only	3
Loesche [35]	Administration of metronidazole only	3, 4
	Surgical intervention	
Loesche 1992 [51]	Administration of metronidazole only	1, 3
	No PPD data presentation as means ± standard deviation	
Loesche 1993 [52]	Administration of metronidazole only	1, 3
	No PPD data presentation as means ± standard deviation	
Lu 2012 [53]	No PPD data presentation as means ± standard deviation	1
Lundstrom 1984 [54]	no adequate control group	2
Magnusson 1984 [8]	No administration of metronidazole	3
Mombelli 2005 [55], Giannopoulou 2006 [84]	Administration of retraction chord, PrefGel® and PGA	1
Moreira 2007 [85]	No group without antibiotics	2
Müller 1986 [86]	Administration of metronidazole only	3
Noyan 1997 [87]	no adequate control group	2
Palmer 1998 [88]	Administration of metronidazole only	3
Palmer 1999 [89]	Administration of metronidazole only	3
Re 1988 [90]	No administration of metronidazole	3
Sigusch 2000 [56]	no adequate control group	2
Sigusch 2001 [57]	no adequate control group	2
Soder 1990 [91]	Administration of metronidazole only	3
Soder 1999 [61]	Surgical intervention	4
Sterry 1985 [92]	Surgical intervention	4
Tinoco 1998 [93]	Surgical intervention	4
Varela 2011 [94]	Same data as Heller [45]	-
Vergani 2004 [95]	no adequate control group	2
Winkel [48]	Administration of metronidazole only	3
**Studies not appliable to the meta-analysis**		
Berglundh 1998 [62]		5
Carvalho 2004 [63]		1
Casarin 2012 [96]		1
Goodson/Mdala 2012 [97]		1
Haffajee [44]		1
Moeintaghavi [38]		5
Ribeiro [39]		1
Rooney [46]		1

1 – missing PPD values as means ± std.

2 – no adequate control group.

3 – no combined administration of amoxicillin and metronidazole.

4 – surgical intervention.

5 – time point of re-examination.

### Study heterogeneity and study characteristics

In some studies smokers were excluded [61–64], one study included only smokers [65] while others [66–75] included both or even did not report on the smoking status of their study population.

Generally, periodontitis cases were classified as generalized chronic or aggressive periodontitis or were not further classified. Not every study reported explicitly to what extent oral hygiene instructions were given before treatment. Frequency and method of supragingival cleaning remains unclear in some studies [67, 69, 70, 73–75]. Test- and control interventions were performed either as full mouth or quadrant-wise treatments with either hand instruments, ultrasonic devices or both. All studies used local anesthesia during subgingival cleaning. Prescribed antibiotics varied in concentration (375–500 mg for amoxicillin and 250/400/500 mg for metronidazole, three times a day each) and the period of intake (7, 10 or 14 days). Different modes of controlling the drug adherence were described. The post-interventional care varied in terms of the use of antiseptic solutions like chlorhexidine of different concentrations and pharmacological forms (gel, mouth washes) and concentrations (0.1/0.12/0.2/1.0%). The periods of investigation varied from 3 to 24 months (Table 2).

**Table 2:** **Study description**

Autor, year of publication	Population	Diagnosis	Treatment prior to intervention	Intervention test/control	Intervention	Control	Parameter assessment	Invest. period	Maintenance	Exclusion	Smokers	Mean age, gender
**Aimetti** [36]	n = 39 systemically healthy; ≥20 teeth excluding teeth indicated for extraction and ≥2 sites around at least 12 teeth with CAL and PD ≥6 mm	gen. aggr. periodontitis	Supragingival Sc and polishing, ohi including Bass technique and id cleaning, tongue 1x/d	1w after screening: OSFMD, SRP in 2 sessions within 24 h (Us), no time limit, dorsum brushing with 1% CHX gel, mouth rinse 2x/d 0.2% CHX, pharynx sprayed (4x tonsil) with 0.2% CHX spray, all pockets irrigated 3x within 10 min with 1% CHX Gel, repeated 8d later; for 2 m: 0.2% CHX 2x/d, tonsil spray 2x	n = 19 OSFMD + **A 500 mg + M 500 mg** 3x/d for 7d	n = 20 OSFMD + Placebo	Presesence of plaque BOP PD Rec CAL (PD + Rec) at 6 sites around all present teeth BL, 3 m, 6 m	6 m	Every 2d to reminder to take medication; no control of empty bottles; check CHX staining, ohi reinforced, full-mouth supraging debridement and professional cleaning on a 2w intervall in first 6w and every 2 m up to 6 m evaluation	Medical disorders or consumption of drugs affecting periodontal status, AB therapy within last 6 m, long-term administration of antiinflammatory drugs, periodontal treatment in previous 6 m, pregnancy, lactation	Excluded	Age T: 36.3 ± 3.2 C: 35.7 ± 2.8 Gender T: 58% f C: 50% f
**Cionca** [29]	n = 47 (4 drop-outs) systemically healthy, ≥12 teeth, no orthodontic appliances, no fixed prostetics, no implants, ≥4 teeth with PD >4 mm, CAL ≥2 mm + rx bone loss.	adult chronic periodontitis	supraging Sc, ohi after 10d: check oral hyg and re-instruction	FM debridement in 2 visits within 48 h: subgingival ScRp: Us, then Gracey, 0.1% CHX, at home 0.2% CHX for 10d 2x/d at the end of final treatment: medication Parallel design	n = 23 **M 500 mg + A 375 mg 3x/d** 7d	n = 24 Placebo	GI, PD, BOP, REC (on 6 sites of teeth with PD >4 mm at BL); Plaque (6 sites, all teeth); microbiological sample at BL, 3 m, 6 m	6 m	10d post-treatment: compliance control, bring back the medication remained; recall 1w, 3 m, 6 m after medication: ohi reinforced, supragingival calculus removed	Systemic diseases, pregnancy, lactation, systemic AB within last 2mt, use of NSAID, periodontal treatment within last year	Recorded	25-70y
**Feres 2012** [41]	n = 118 (at 6 m 5 drop-outs, at 12 m 17 drop-outs); good gen health; ≥30y; ≥6 teeth with at least 1 site each with PD and CAL ≥5 mm, at least 30% of sites with PD and CAL ≥4 mmm and BOP	gen chronic periodontitis	ohi, same dentifrice (Colgate total)	SRP in 4–6 session for 1 h each, manual instruments; entire oral cavity within 14d	Immediately after first session of SRP n = 39 **M 400 mg 3x/d 14d** CHX +/- n = 39 **SRP + M 400 mg + A 500 mg 3x/d 14d** CHX +/- CHX: rinse 15 ml 0.12% CHX or placebo for 1 min 2x/d 2 min	n = 40 SRP + Placebo CHX +/-	visible plaque gingival bleeding BOP Suppuration PD (at 6 sites) CAL (at 6 sites) Hu-Friedy BL, 3 m, 6 m, 12	12 m	At 3 m, 6 m, 12 m; at the end of each week of medications asked to return bottles/flasks; questionnaire about self-perceived side effects; calling subjects every 2d to monitor AB-compliance	Previous subgingival periodontal therapy, pregnancy, nursing, systemic diseases affecting periodontal status, long-term administration of anti-inflammatory drugs, need for AB-premedication for routine dental therapy, AB therapy within last 6 m, allergy to M, A or CHX	Excluded	C: 45.8 ± 8.54y 12 m/28f M: 43.4 ± 8.26y 15 m/24f MA: 46.3 ± 8.59y 17 m/22f
**Heller** [45]	n = 31 (4 drop-outs) ≥16 teeth; ≥4 sites on different teeth with PD ≥6 mm, CAL ≥5 mm, moderate to severe bone loss and BOP	gen aggr periodontitis	ohi in 2 weekly sessions, aim <20% PlI	**Phase I:** FM debridment with Us 2x1h, irrigation of all pockets with a gel 0.2% CHX within 24 h, rinse and gargle 2x/d with 0.12% CHX, brush tongue 2x/d with gel for 45d. After last session assigned to group test or control. **Phase II:** quadrant scaling manual 1 h within 4-6w; irrigation of pockets: 0.2% CHX Parallel design	n = 18 **A 500 mg + M 250 mg 3x/d** 10d	n = 17 Placebo	Clin exams at BL, 3 m, 6 m 6 sites per tooth PD and CAL BOP + or – Plaque GI Suppuration BL, 3 m, 6 m	6 m	3 m follow up visit: ohi reinforcement, FM supragingival cleaning; **sites with PD > 4 mm and BOP were reinstrumented unter LA**	Allergy to penicillin, M or CHX, systemic diseases affecting periodontal status, longterm-used antiinflammatory medication, periodontal treatment or AB in last 6 m, pregnancy, lactation	No data	18-39y
**Matarazzo** [40]	n = 43 (2 drop-outs) ≥15 teeth, ≥6 sites with PD 5-7 mm and CAL 5-10 mm	chronic periodontitis	Clinical and mikrobiological monitoring, FM supragingival scaling, ohi, same toothpaste (Colgate total)	SRP in 4-6x appointments 1 h each within max of 21d, AB therapy initiated at first SRP visit	n = 14 SRP **A 400 mg M 400 mg 3x/d** 14d n = 14 SRP + **M 400 mg + A 500 mg 3x/d** 14d	n = 15 SRP + Placebos	Visible plaque gingival bleeding BOP Suppuration PD CAL at 6 sites, Hu-Friedy BL, 3 m	3 m	Had to bring tubes containing medication at every SRP visit (pills were counted); calling every 4d to monitor compliance	Aggr periodontitis, pregnancy, lactation, periodontal or AB therapy in previous 6 m, systemic conditions affecting progression of periodontal disease, longterm administration of antiinflammatory drugs, need for AB coverage for routine dental therapy, allergy to M and/or penicillin	Only smokers at least 10 cig/d for last 5y	All >30y **age** SRP: 40.5 ± 8.2 y SRP + M: 40.8 ± 5.1 y SRP + M + A: 42.8 ± 7.1 y **gender** SRP: 7/8 m/f SRP + M: 7/8 (6/8) m/f SRP + M + A: 7/8 (6/8) m/f
**Mestnik** [37]	n = 30 systemically healthy, ≥ 20 teeth, ≥6 permanent teeth including incisiors and/or first molars with PD and CAL ≥5 mm and ≥6 teeth other that first molar and incisors with at least one site each with PD and CAL ≥5 mm, familiar aggregation	gen aggr periodontitis	FM supraging Sc and ohi, same Dentrifice (Colgate total)	FM SRP in max 6 sessions 1 h within 14d, manual instruments; rinsing with 15 ml 0.12% CHX 1 min 2x/d 60d. AB and CHX rinses starts immediately after 1. session of mechanical instrumentation.	n = 15 SRP **M 400 mg + A 500 mg 3x/d** for 14d	n = 15 SRP und Placebo	Visible plaque Gingival bleeding BOP Suppuration PD CAL at 6 sites BL, 3 m	3 m	1x/w bring packs back, check compliance; calling every 2d to monitor compliance	Previous subgingival periodontal therapy, smoking, pregnancy, systemic desease affecting progression of periodontal disease, long-term administration of anti-inflammatory medication, need for AB coverage for routine dental therapy, AB therapy in previous 6 m, allergy to CHX, A, M	Excluded	≤30y Age T: 26.8 ± 3.9 y C: 27.6 ± 3.5 y Gender T: 6/9 m/f C: 4/11 m/f
**Mombelli [** [24]	n = 82 systemically healthy, ≥12 scorable teeth (no 3th molars, no teeth with orthodontic appliances, bridges, crowns or impl), diagnosif of periodontitis with ≥4teeth with PD >4 mm, CAL ≥2 mm + rx evidence of bone loss	chronic periodontitis (untreated moderate to advanced)	supragingival cleaning, ohi, recalled to assure good oral hygiene	SRP within 48 h usually in 2 sessions with Us, Gracey curettes, then irrigated the pockets with 0.1% CHX; at home rinse 2x/d for 10d with 0.2% CHX	n = 22 Aac - n = 22 Aac + **SRP + 500 mg M + 375 A 3x/d for 7d**	n = 19 Aac - n = 19 Aac + SRP + Placebo	GI PD REC BOP Suppuration PS (6sites of all teeth) on 6 sites of each tooth with PD >4 mm at BL BL, 3 m	3 m	1w after treatment: return any medication left	systemic illnesses, pregnancy, lactation, AB taken within previous 2 m, use of NSAIDs, confirmed or suspected intolerance to 5-nitroimidazole-derivates or A, subgingival SRP or surgical periodontal therapy in the last year	Recorded	25-70y T: 21 females 17 smokers C: 20 females 12 smokers
**Preus** [99]	n = 180 (4 drop-outs) no prior systematic periodontal treatment, after pre-study hygiene phase ≥5sites with PD ≥5 mm remained,	moderate to severe oeridontitis	3 m hygiene phase: ohi, supraging. Sc, necessary extraction, endodontic treatment, filling and temporary prosthetics done by general dentist	Gr 1 + 2: FM SRP (FMD) completed within a single workday in 2x 65 min, 2 h apart, SRP completed in 2x 65 min each, 21d apart All: rinsed for 1 min with 10 ml 0.2% CHX; Us, hand and rotating instruments, first 1. + 4.Q, second 2. + 3 Q; air-flow or pumice paste; contact points flossed, sulci + pockets filled with a 1% CHX gel Home care instructions in brushing teeth and tongue and rinse with 0.2% CHX: every morning for 9d parallel design	n = 44 FMD + **M 400 mg 3x/d 10d** n = 45 SRP + **M 400 mg 3x/d 10d**	n = 45 FMD + Placebo n = 46 SRP + Placebo	PD CAL Plaque yes no BOP 4 sites of all teeth BL, 3 m, 12 m	12 m	After 7d a quality control of the scaling and 7w later (8w posttreatment) reinforcement of ohi Supportive treatment sessions at 3, 6, 12 m after active therapy	Syst diseases known to be associated with perio, contunuous medication known to affect perio, allergy to M	Recorded	35-75y Gr1: 53.7 ± 7.6y 43.5% women 47.8% current smokers 93.5% current/former smokers Gr2: 55.1 ± 7.9y 62.2% women 53.3% current smokers 75.6% current/former smokers Gr3: 56.8 ± 8.3y 43.5% women 63.0% current smokers 84.8% current/former smokers Gr4: 54.9 ± 8.5y 51.1% women 57.5% current smokers 95.7% current/former smokers
**Sigusch 2001** [57]	n = 25 (M) systemically healthy, average of 16 sites with PD >8 mm and intrabony lesion at ≥ 1–5 teeth over two-thirds of the root length	gen rapidly progressive periodontitis		1.step: SRP in 4–5 sessions including ohi 2.step: 3w later FM Rp in 1 or 2 2 h -sessions with no more than 2d between sessions, wound dressing	First dose immediately after the 2.step n = 12 Doxy n = 15 **M 2x500mg**, 8 days n = 11 Clindamycin	n = 10	BL, 3w after SRP (first step), 6 m, 24 m after enhanced Rp (second step) PI Sulcus BI PD CAL Suppuration at six sites per tooth	6 m, 24 m	Recall sessions every 4-6w for 6 m and every 12w thereafter	AB therapy within last 6 m, history of recurrent disease other than periodontitis; flap surgery in the past 6y	Excluded, unless they had stopped smoking 2 m prior to therapy	Mean age: 32.4y Gender m/f 20/28
**Winkel** [48]	n = 21 > 25y, ≥3 natural teeth in each quadrant; ≥1 site with PPD >5 mm with BOP and rx evidence of bone loss in each quadrant	gen adult periodontitis		FM initial perio dontal treatment: SRP and ohi, 3–6 sessions of 1 h, at each session ohi reinforced. 6w after last SRP session: FM check up and SRP if PD > 3 mm and BOP. Ohi and reinforcment	n = 10 **A 500 mg + Clavulanic acid** 125 mg 3x/d for 10d	n = 11 SRP	PPD CAL constant force probe, Brodontic PI GI BOP	BL, 3 m, 6 m, 9 m, 12 m	Recording time of intake medication on a diary; returned the unused pills, call 2w after the end of the medication	hypersensitivity toward ß-lactam agents, professional SRP or surgical periodontal therapy in the past and AB therapy 6 m prior to treatment, pregnancy, lactation, planing pregnancy, systemic disease, acute necrotising periodontitis; use of non-steroidal anti-inflammatory drugs	Smoker: T: 5 C: 5	Gender m/f T: 2/8 C: 4/7 age T: 49y (36–66) C: 39y (28–47)
**Winkel** [49]	n = 49 ≥ 3 natural teeth in each quadrant; ≥1 site in at least 3 of the 4 quadrants with PPD >6 mm and CAL ≥3 mm, BoP and radiographic evidence of alveolar bone loss	no data		FM SRP in 3–6 sessions of 1 h, at each session ohi reinforced 6w after 1st session: recall for FM check-up and SRP at sites with PD >3 mm and BOP, including ohi reinforcement. on this day: medication	n = 23 **A 375 mg + M 250 mg** 3x/d, 7d	n = 26	PI PPD BI (bleeding index) CAL	BL, 6 m	Ohi reinforcement at every SRP session and after 6w return med after 7d	SRP or surgical periodontal therapy; periodontal AB therapy 6 m prior to the initiation of the study; pregnancy, lactating or planing pregnancy; systemic ; acute necrotising periodontitis; use of NSAIDs, use of mouthrinses	Recorded. smoker = also if had stopped within the last year T: 14/23 C: 18/26	mean age 42y (28–63) mean age T: 45y (32–63) mean age P: 40y (28–55) gender m/f T: 11/12 C: 10/16
**Xajigeorgiou** [50]	n = 43 (4 drop-outs) n = 33 (A + M) ≥20 teeth,	gen aggr periodontitis (with amiliar aggregation)	BL sampling of subging plaque and FM clinical recordings	Ohi and FM SRP, 4 visits; f PI ≤20 continued after the 6w; debridement	n = 10 SRP **M 500 mg + A 500 mg** 3x/d, 7d n = 12 SRP+ **M 500 mg** 3x/d, 7d	n = 11 SRP	BL 6w after SRP 6 m PD (Hu-Friedy) AL BOP at six sites	6 m	reinforcement of ohi biweekly from BL to 6w	AB intake in the last 3 m, AB allergies, periodontal treatment during previous 12 m, pregnancy, lactation	Recorded MA 3/10 M 5/12 C 4/11	Age 22-49y M + A 38.9 ± 8.7 M 40.9 ± 4.6 C 37 ± 5.6 Gender m/f M + A 5/5 M 4/6 C 6/5

A → Amoxicillin.

AB → antibiotics.

aggr → aggressive.

AL → Attachment level.

BOP → Bleeding on probing.

CAL → clinical attachment level.

CHX → chlorhexidine.

d → day/days.

FM → full mouth.

FMPS → Full Mouth Plaque Score.

FMBS → Full Mouth Bleeding Score.

GBI → gingival bleeding index.

gen → generalized.

GI → Gingival index.

id → interdental.

io → intraoral.

loc → localized.

m → month(s).

M → Metronidazole.

m/f → ratio males/females.

NSAID → non steroid anti-inflammatory drugs.

ohi → oral hygiene instruction.

OSFMD → One Stage Full Mouth Disinfection.

PD → probing depth.

PI → plaque index.

pol → polishing.

PS → Plaque score.

REC → Recession of the gingival margin.

rx → radiographic.

Sc → scaling.

SRP → Scaling and Root planing.

Us → Ultrasonic device.

VPI → visible plaque index.

w → week(s).

### Quality assessment

The quality assessment is presented in Table 3. If the method of randomization, concealment or the blinding of the examiner was clearly described, the quality was rated as “+” if it was claimed that randomization, concealment or the blinding was performed but no information about the mode of performance was provided the rating was “(+)” and if no concealment or blinding was stated, the rating was “–”. Based on this rating, study quality was assessed as moderate to high.

**Table 3:** **Quality assessment**

Author, year of publication	Method of randomisation [+/(+)/-]	Concealment [+/(+)/-]	Blinding of the examiner [+/(+)/-]
**Aimetti** [36]	+	+	+
	Computer generated list		
**Cionca** [43]	+	+	+
	Computer generated list		
**Ferres 2012** [41]	+	+	+
	Computer generated list		
**Heller** [45]	+	+	+
	Computer generated list		
**Matarazzo** [40]	+	+	+
	Computer generated list		
**Mestnik** [37]	+	+	+
	Computer generated table		
**Mombelli** [24]	+	+	+
	Computer generated list		
**Preus** [99]	+	+	+
	Computer generated table		
**Sigusch 2001** [57]	(+)	-	(+)
**Winkel** [48]	(+)	-	+
**Winkel** [49]	(+)	-	+
**Xajigeorgiou** [50]	+	-	+
	Randomization table		

+ modality explained.

(+) claimed without further explanation.

- not reported.

### Study outcomes

**Figure 3:**
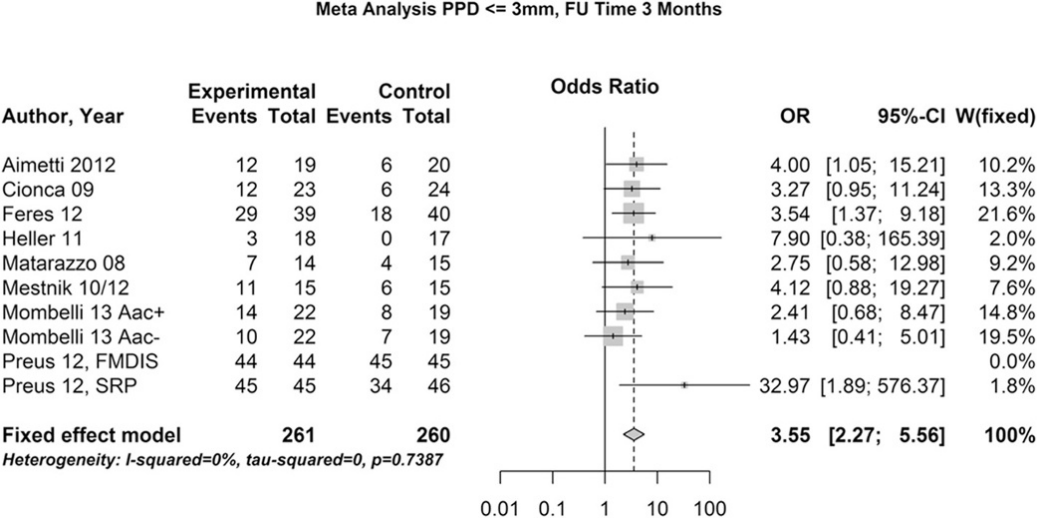
**Meta-analysis of the chance for pocket closure after 3 months.** OR – odds ratio, 95-CI – 95% confidence intervall, w – weight, p – level of significance.

**Figure 4:**
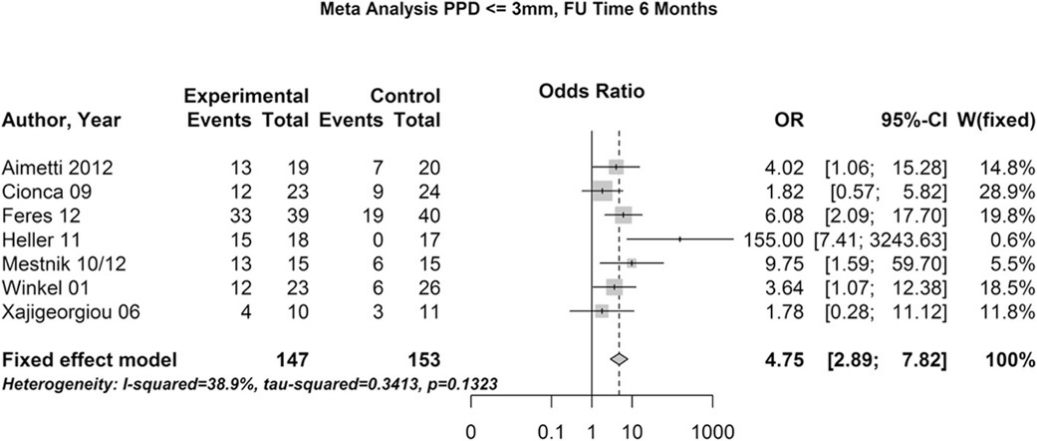
**Meta-analysis of the chance for pocket closure after 6 months.** OR – odds ratio, 95-CI – 95% confidence intervall, w – weight, p – level of significance.

For the re-evaluation time points we performed two meta-analyses, one at 3 months after treatment and one after 6 month, which included 10 and 7 studies with a total of 521 and 448 patients, respectively.The meta-analyses revealed that the use of the combination of amoxicillin and metronidazole together with SRP increased the chance of pocket closure by a factor of 3.55 three month after the therapy (Figure 3) and a pronounced 4.43 fold chance six month after the treatment (Figure 4).

We found that it was not possible to calculate the risk estimation for residual pockets exceeding 5 mm, as there was an estimated risk for residual pockets over 5 mm of 0 for both treatment types, which rendered the comparative calculation impossible.

The estimated percentage for pertinent pockets exceeding 3 and 5 mm for the additional use of antibiotics and for SRP alone is presented in Table 4a (at time point 3 months) and b (at time point 6 months).

**Table 4:** **Percent of persisting pockets deeper than 3 mm and 5 mm**

Paper	PPD > 3 mm		PPD > 5 mm	
	Test (AB type = 1)	Control (AB type = 0)	Test (AB type = 1)	Control (AB type = 0)
**a) At 3 months follow up**				
Aimetti [36]	37	70	0	0
Cionca [29]	52	75	0	0
Feres 2012 [41]	26	55	0	0
Heller [45]	83	100	0	0
Matarazzo [40]	50	73	0	0
Mestnik [37, 53]	27	60	0	0
Mombelli [24] Aac+	36	58	0	5
Mombelli [24] Aac-	54	63	0	5
Preus 2013 [99], FMDIS	0	0	0	0
Preus 2013 [99], SRP	0	26	0	0
**b) At 6 months follow up**				
Aimetti [36]	32	65	0	0
Cionca [29]	42	63	0	0
Feres 2012 [41]	15	53	0	0
Heller [45]	17	100	0	0
Mestnik [37, 50]	13	60	0	0
Winkel [49]	52	76	0	0
Xajigeorgiou [50]	60	72	0	0

AB type 1 – systemic antibiotic administration.

AB type 0 – no antibiotic administration.

Aac + - Regarding Evaluation from subgroup positiv for *A. actinomycetemcomitans.*

Aac- - Regarding Evaluation from subgroup negative for *A. actinomycetemcomitans.*

For two additional studies [62, 71] with data given for both the means and standard deviations and the exact proportion of residual pockets, we performed the same estimation like for the included studies in order to re-validate the statistical model (Table 5). The comparison of published and calculated ratios show a qualitative accordance. However, some subgroups showed considerable differences in size.

**Table 5:** **Comparison of published and calculated OR for the use of antibiotics for studies providing both means and standard deviations and percentages of residual pockets**

Study	Cut-off [mm]	Data	3 m	6 m
Rooney [46]	3	published	2.8	2.6
	3	calculated	7	7
Mestnik [37, 50]	5	published	1.7	2.2
	5	calculated	1	2.2
Rooney [46]	6	published	11.3	9.5
	6	calculated	3.4	2.2

## Discussion

This study aimed to estimate the chance of pocket closure or avoidance of surgical therapy after non-surgical periodontal treatment comparing the treatment with and without the additional use of the combination of the amoxicillin and metronidazole. Other than in conventional systematic reviews and different to the data presentation recommended by the PRISMA statement [76], this review did not present the differences by means and standard deviations, but estimated the likelihood for the attainment of clinical relevant surrogate parameters. We believe that this kind of data presentation provides easier and clinically more relevant interpretations of the clinical effectiveness, as in periodontal treatment the main target is the reduction of pockets below a cut-off pocket depth of less than 3 mm or not exceeding 5 mm [26]: The first benchmark indicates that the pockets are “closed” with no further treatment needs, whereas the second benchmark indicates the avoidance of specific needs for a surgical intervention, which is classically still indicated if pockets of 6 mm depth and deeper persist after treatment due to their significantly enhanced risk for disease recurrence [26].

This study clearly elucidated an enhanced chance for pocket closure when antibiotics were used in combination with mechanical root surface debridement. On the other hand, the calculations could not be performed for the case of a cut-off value > 5 mm. This implied that the statistical model indicated a 100% elimination of pockets > 5 mm for both the treatment with and without the use of antibiotics. This fact depicts a shortcoming of the performed statistical estimation, as the single studies in fact reported isolated residual pockets.

In all the included studies, we assumed a normal distribution of the data [77]. With relatively small case numbers, this expectation might have distorted the calculated results to some extent. However, the effect of possible statistical misclassification was likely to be similar in test and control group due to the randomization of the treatment allocation of the studies, thus limiting the disturbing bias again.

We tried to verify the adaptability of the statistical model using the calculation in studies that provided both, means and standard deviations and the exact distribution of residual pockets of either > 3 mm or > 5 mm depth. In the data of one study group [62, 78] we found a good correlation of true and calculated results. However, in another the true and estimated values varied to a greater extent [71], despite the fact that important factors such as sample size were comparable.

The calculation model has been previously published and more studies using this analysis methodology have been demanded [32]. With the present data, its applicability can be better understood and its limitation to studies with higher numbers of participants appears recommendable. In conclusion, the data presentation of the exact distribution of the pocket depths over 3 and 5 mm – as already presented in the actual literature – should be provided in future studies as it was done in the classical studies as well. However, such a request needs time to push through and as long as this claim is not generally implemented, the proposed statistical model offers a useful alternative method to combine and compare study results in such a way.

The pronounced effect of the antibiotics during healing after the first three months as compared to healing after SRP alone is well reflected well in our analysis: For pocket closure, there is an enhanced chance after 6 month if antibiotics had been used. This finding is in accordance with the existing literature [30, 79, 80].

A large heterogeneity existed for the included data: Smoking status, diagnosis of aggressive or chronic periodontitis and the detailed treatment scheme used in the studies showed substantial variations. With the cumulative analysis there was a certain risk of comparing apples with oranges. However, this approach offered the possibility to generate a universal conclusion on the antibiotic treatment of periodontitis, regardless of which patients were treated with which protocol. Furthermore, and another limitation of our approach, we could not include important studies assessing the issue of interest because of the way on which their data was presented: Several authors presented their data well and even with the similar aim to refer to distributions of specific benchmark values, but unfortunately other cut-off values than ours were chosen, which rendered a comparison impossible.

With 10 and 7 included studies for the time points 3 and 6 months after treatment, respectively, only a relatively small number of studies dealing with the issue of antibiotics in periodontitis treatment could be included. Zandbergen et al. assessed a body of 24 studies in a classic review [30]. However, aiming to perform a meta-analysis they could only calculate the overall change of PD and CAL for SRP in combination with antibiotics. Neither a direct comparison to the treatment without antibiotics, nor an estimate of the treatment success in terms of pocket closure or avoidance of surgical therapy was possible in their review. These aspects, however, are important to both the practitioner and the patient.

The benefits of antibiotic treatment always have always to be balanced against their possible adverse reactions. For amoxicillin allergic skin reactions, joint swelling and – in few cases – anaphylactic reactions are well documented [81]. Metronidazole has frequently been reported to cause – among other discomforts and indispositions - nausea, diarrhea and headache [82]. The dimension of the potential risk of causing resistant strains against these antibiotics must also be kept in mind, even if the discussion about this issue is controversial in the current literature [25, 82, 83]. For the clinician a clear prediction of the benefits of adjunctive antibiotic therapy in therms of residual treatment needs after non-surgical treatment might be an easier and better tool for the consideration of a possible antibiotic prescription than rather abstract means and standard deviations as predominantly presented in conventional reviews.

## Conclusion

Using a distribution based statistical approach, it was shown that there is a clear benefit in terms of an enhanced chance for pocket closure by co-administration of the combination of amoxicillin and metronidazole as adjunct to non-surgical mechanical periodontal therapy. However, based on the currently available data a potential benefit in terms of the possible avoidance of surgical interventions could not be delineated.

## Appendix

The following formula for the calculation of the probabilities P(X”x) for clinical success, ie, for pockets >3 mm was used:

F(X)(x) describes the cumulative distribution function,  the mean,  the standard deviation and x the cutoff value, eg 3 mm or 5 mm.

For the expression:

One can determine the probabilities by consulting normal distribution tables [58].  stands for the distribution function of the standard normal distribution. The probability values in the Tables published by Stahel are only valid for standard normal distribution. For example, for a group of nine patients, the mean pocket depth at followup is 3.53 mm with a standard deviation of 0.62. Therefore, for:

We get ((3.00 - 3.53)/0.62) = -0.85. The probability for pockets ≤ 3 mm is 0.20, as derived from the Table in Stahel [58]. Hence, the number of patients with pockets ″Accordingly, the number of patients with pockets ≤ 3 mm is 9 × 0.20 and the number of patients with pockets >3 mm is 0.80×9 [32].
